# Translation and validation study of the Polish version of the Brief Hepatitis C Knowledge Scale

**DOI:** 10.1371/journal.pone.0235764

**Published:** 2020-07-09

**Authors:** Olga Tronina, Joanna Gotlib, Piotr Małkowski, Mariusz Jaworski, Mariusz Panczyk

**Affiliations:** 1 Department of Transplantation Medicine, Nephrology and Internal Medicine, 1^st^ Faculty of Medicine, Medical University of Warsaw, Warsaw, Poland; 2 Department of Education and Research of Health Sciences, Faculty of Health Sciences, Medical University of Warsaw, Warsaw, Poland; 3 Department of Surgical and Transplantation Nursing and Extracorporeal Therapies, Faculty of Health Sciences, Medical University of Warsaw, Warsaw, Poland; Murcia University, SPAIN

## Abstract

**Introduction:**

Chronic hepatitis C (HCV), considered by the World Health Organization as one of the greatest epidemiological health hazards, often with asymptomatic clinical course and one which, due to scanty knowledge, remains a crucial risk factor of serious chronic HCV infection complications. The purpose of this study is to validate the psychometric properties of the Polish version of the validated Brief Hepatitis C Knowledge Scale (BHCKS_PL), developed by Balfour in 2009.

**Methods:**

The study, conducted from May to July 2018, included 246 persons (68,69% females), divided into four subgroups: patients (n = 86), nursing students (n = 74), medical students (n = 28), healthcare workers (nurses and doctors; n = 58).

The 19-items questionnaire contained questions designed to assess general knowledge regarding hepatitis C and the transmission risk factors.

**Results:**

An evaluation by means of multiple comparisons in pairs showed that there were significant differences in the knowledge level between the group of patients and the group of nursing students (Mdn: 14.0 vs 11.0, z = 7.713, P<0.001), and between students of medicine (Mdn: 16.0 vs 11.0, z = 0.339, P<0.001) and healthcare workers (17.0 vs 11.0, z = 11.447, P<0.001). Moreover, significant differences were observed between the groups of students of nursing and medicine (Mdn: 14.0 vs 16.0, z = 3.646, P = 0.002) and healthcare workers (Mdn: 14.0 vs 17.0, z = 4.117, P<0.001). No significant differences in the knowledge level between the students of medicine and healthcare workers were observed (z = 0.377, P = 1.000).

**Conclusions:**

The completed validation suggests good BHCKS_P psychometric characteristics with the internal consistency convergent and known-groups validity. The questionnaire can be used in educational practice. The obtained results of the measurement provide information about the studied person based on the total score.

## Introduction

The goal of eradicating the hepatitis C virus (HCV) infections is one of the greatest challenges that the World Health Organisation and the European and world infectious disease and hepatologist societies are currently facing [1].

Among the global population there are 71 million people infected with HCV, but due to slight manifestations of the infection as many as 80% of the infected are not aware of it [2]. After the virus enters the host’s cells and the acute inflammation phase is over, a chronic inflammation state occurs in 75–85% of cases. After 20–30 years of chronic inflammation, cirrhosis develops in 10–20% of cases [3].

An additional problem is the lack of possibility to vaccinate against new HCV infections, which could become a milestone in the elimination of this health and life threatening disease [4]. Every year almost 400,000 people die due to complications caused by chronic infection, cirrhosis and hepatocellular carcinoma [2].

The high risk group includes drug users, prisoners, persons with risky sexual habits, as well as patients undergoing repeated invasive medical procedures and recipients of blood products, especially in the case of transfusions before year 1992 [5].

A special group of patients susceptible to HCV infection are patients with a chronic kidney disease and undergoing hemodialysis, for which the risk of being infected with HCV is directly linked to sanitary neglect on the behalf of the personnel overseeing the procedures of renal replacement treatments [6, 7, 8].

The webpage of the World Health Organisation published a document adopted from the summary of the 69th session of the World Health Assembly held in June 2016, ‘Global Health Sector Strategy on viral hepatitis 2016–2021’, aiming to eliminate the viral infections by year 2030 [1, 9].

Although these plans refer to all five hepatotropic viruses, special attention is paid to hepatitis B and C, as they are responsible for the highest number of fatal complications [2]. According to the set goals, by year 2030, 90% of infected will be diagnosed, new infection count will be reduced by 90%, fatal complications related to HBV (hepatitis B virus) and HCV virus infections will be reduced by 65% and 80% of the infected will receive antiviral treatment [1, 10]. The registration of drugs with direct antiviral effects, with their >90–95% effectiveness and high safety levels of treatment make these goals more realistic [11, 12]. In 2016 as many as 1.76 milion people received HCV antiviral treatment, although this is still only 13% of the total requirement for treatment [13].

In Poland in 2012 the KIK/35 project was initialised, called ‘HCV infection prevention’ as a part of the Switzerland-Poland cooperation programme and is aimed to educate and raise social awareness on HCV epidemiology in Poland [14].

The social information campaign conducted as a part of this project was supposed to increase awareness on the infection issues, complications, transmission risks, but most of all on the possibilities of preventing HCV infections from spreading.

The improvement of social awareness and knowledge not only increases the percentage of people undergoing a screening test, but also increases the number of people who try to increase diagnostics and begin antiviral treatment.

Health education programmes can be successfully conducted only when they are preceded by reliable and thorough analyses of the target group’s knowledge level. An analysis of papers available in the literature on the knowledge level of HCV among different groups reveals that the majority of studies are carried out using proprietary questionnaires, which makes comparison of the study results impossible [15–20]. A vast majority of the tools is not validated: their authors do not quote indices of repeatability or measurement reliability, which may challenge the validity of the knowledge level measurement in the studied groups.

The only standardised questionnaire available in the literature intended for measuring knowledge on HCV is the Brief HCV Knowledge Scale published in 2009 and developed by Balfour, et al. [21]. Further, no standardised scale for measuring the knowledge of HCV has been published in the Polish literature [22, 23]. With regard to the validity of the HCV infection issue in Poland, there is a need to develop a scale for examining HCV knowledge levels. A reliable scale measuring HCV knowledge level can become an effective tool for a quick analysis of knowledge levels for health care specialists such as physicians, nurses or health educators, as well as for patients.

The purpose of this study was to evaluate the psychometric properties of a Polish version of the validated Brief Hepatitis C Knowledge Scale (BHCKS_PL).

## Materials and methods

### Design

Cross-sectional validation design: a diverse sample of 246 participants who were recruited through convenience sampling. The study was conducted from May to July 2018.

### Participants

The study involved 246 people, including 169 (68.69%) women, 71 (28.86%) men and 6 persons (0.2%) who did not respond to the question regarding sex. All persons participating in the study gave their consent and could be classified as a member of one of the subgroups listed below. The study group consisted of four subgroups: (1) patients (n = 86); (2) nursing students (n = 74); (3) medical students (n = 28); (4) healthcare workers (n = 58). The average age was 47.9 years (min. 20, max. 69, SD = 10.32, CV = 26.2%). The groups of participants vary in age, especially in relation to the group of students, which was significantly younger (usually < 30 y.o.) than the groups of patients and healthcare workers (majority > 30 y.o.). In relation to sex, the studied group was clearly dominated by women, which is a result of the fact that among Polish students of nursing, 95% are women, and a similar domination of the female sex can be seen among healthcare workers in the units were the study was conducted.

The study was conducted in June 2018. Participation in the study was voluntary and anonymous, and the respondents were asked to fill in the questionnaires on their own. The paper-and-pencil technique was used. Detailed characteristics of the studied group are presented in Table 1.

**Table 1:** Participant characteristics.

	Patients	Nursing students	Medical students	Healthcare workers
**N**	86	74	28	58
**Gender**				
female	32 (37.2)	71 (95.9)	19 (67.9)	47 (81.0)
male	48 (55.8)	3 (4.1)	9 (32.1)	11 (19.0)
missing data	6 (7.0)	-	-	-
**Age (M (SD))**	47.9 (14.7)	22.3 (2.0)	24.4 (1.4)	44.2 (9.9)
**Place of residence**				
countryside	22 (25.6)	22 (29.7)	4 (14.3)	5 (8.6)
village (population up to 50 thousand)	18 (20.9)	3 (4.1)	1 (3.6)	3 (5.2)
small town (51–200 thousand inhabitants)	15 (17.4)	7 (9.5)	1 (3.6)	1 (1.7)
large town (201–500 thousand inhabitants)	6 (7.0)	1 (1.4)	0 (0.0)	3 (5.2)
city >500 thousand inhabitants	21 (24.4)	41 (55.4)	21 (75.0)	46 (79.3)
missing data	4 (4.7)	-	1 (3.6)	-
**Education**				
primary/lower secondary	2 (2.3)			
vocational	16 (18.6)			
secondary	40 (46.5)			
tertiary	23 (26.7)			
missing data	5 (5.8)			
**Profile of education**				
medical	9 (10.5)			
non-medical	54 (62.8)			
missing data	23 (26.7)			
**Employment**				
full-time job	31 (36.0)			
works occasionally	5 (5.8)			
retired/receiving disability pension	36 (41.9)			
does not work	9 (10.5)			
missing data	5 (5.8)			
**Has HIV infection ever been diagnosed?**				
yes	3 (3.5)	0 (0.0)	1 (3.6)	0 (0.0)
no	80 (93.0)	72 (97.3)	27 (96.4)	54 (93.1)
don’t know	1 (1.2)	2 (2.7)	0 (0.0)	2 (3.4)
refusal	2 (2.3)	0 (0.0)	0 (0.0)	2 (3.4)
**Has HCV infection ever been diagnosed?**				
yes	21 (24.4)	0 (0.0)	1 (3.6)	0 (0.0)
no	59 (68.6)	73 (98.6)	27 (96.4)	56 (96.6)
don’t know	4 (4.7)	1 (1.4)	0 (0.0)	1 (1.7)
refusal	2 (2.3)	0 (0.0)	0 (0.0)	1 (1.7)
**Has any of your family members/relatives/friends been diagnosed for HCV infection?**				
yes	28 (32.6)	11 (14.9)	4 (14.3)	30 (51.7)
no	46 (35.5)	56 (75.7)	22 (78.6)	25 (43.1)
don’t know	9 (10.5)	6 (8.1)	1 (3.6)	2 (3.4)
refusal	0 (0.0)	0 (0.0)	0 (0.0)	1 (1.7)

M—mean, SD—standard deviation.

The patients who took part in the study were hospitalised at the Department of Transplantation Medicine, Nephrology and Internal Medicine of the Infant Jesus Clinical Hospital, Medical University of Warsaw (Poland) due to liver cirrhosis of various etiology, including chronic hepatitis C, were qualified for a transplant and were after transplant and hospitalised due to post-transplant complications.

The patients were asked to fill in questionnaires during their check-up appointments in the hospital’s Nephrology and Transplantation Outpatient Clinic.

Another group consisted of students of the Medical University of Warsaw: third-year nursing students at the Faculty of Health Sciences, and fifth- and sixth-year students at the Faculty of Medicine. Students of both majors completed their curriculum courses on infectious disease issues. They filled in the questionnaires during their classes at the Department of Transplantation Medicine, Nephrology and Internal Medicine of the Infant Jesus Clinical Hospital, Medical University of Warsaw (Poland).

The group of healthcare workers included nurses (n = 4) and physicians (n = 54) employed at the Infant Jesus Clinical Hospital (n = 15), Centrum Medyczne Medicover nonpublic medical healthcare centre (n = 14) and Centrum Medyczne LUXMED non-public medical healthcare centre (n = 19). Ten people did not reveal their places of work.

### Ethical considerations

The study was voluntary and anonymous. Prior to joining, each participant was asked to express consent. The purpose of the study, as well as the methods of data storage and analyses, were communicated to the participants in writing. Their written consent to take part anonymously and of their free will was expressed by filling out the survey. Signed copies were archived. They were also informed that the collected data was confidential and would be used only for research purposes. The authors sought approval from the Bioethics Committee of the Medical University of Warsaw to conduct the presented study (approval number: KB/61/2018).

### Instrument

The Brief Hepatitis C Knowledge Scale (BHCKS) was developed by Balfour, et al. [21]. The BHCKS contains 19 items concerning knowledge, in the form of questions with ‘true’, ‘false’ and ‘don’t know’ answers. The answers given by the participants were scored according to the key developed by Balfour, et al. [2] (see details in the S1 Data).

One point was awarded for each correct response. Incorrect responses and responses of “don’t know” were grouped for analysis as “not correct” (no point).

The original 19 items pool was generated based on an extensive HCV literature review, which included scientific articles published in scholarly journals and brochures and educational material obtained from community-based agencies. Item content areas included: knowledge about HCV transmission (e.g. *There is some risk that hepatitis C can be given to someone by snorting cocaine with shared straws*, *rolled money*, *etc*.), prevention (e.g. *There exists a hepatitis C vaccine that can be used to prevent people from getting infected with the hepatitis C virus*) and treatments (e.g. *Successful hepatitis C treatments can result in the hepatitis C virus being completely removed (or cleared) from one’s blood*) [21].

The scale was validated by Balfour, et al. [21] on a group of 406 participants: HCV monoinfected patients (n = 83), HCV/HIV coinfected patients (n = 24), HIV monoinfected patients (n = 128), community healthcare workers (n = 89) and college students (n = 82). The tool was found to have high levels of content and construct validity, as well as reliability (internal consistency and test-retest stability) [21]. The validation results also showed that the 19 items of BHCKS were one-dimensional. Reliability and validity were consistent in patients, healthcare workers and students.

### Procedure

Balfour, the author of BHCKS, has given his permission to carry out a Polish validation of the questionnaire. Linguistic–cultural adaptations and the identification of psychometric properties of the Polish language version of BHCKS were made according to the guidelines developed by Sousa and Rojjanasrirat [24] and the World Health Organization’s ‘Process of translation and adaptation of instruments’ [25]. The translation and validation procedure consisted of four phases.

**Phase I**. The English version of BHCKS in the form published by Balfour, et al. [21] was translated by two independent translators. The first was bilingual with Polish as their native language, fluent in both the original language of the questionnaire and the target language. The translator was not familiar with the terminology included in the questionnaire but had a good knowledge of the common language and the common use of the target language. The other translator was also bilingual, with English as their mother tongue. They were also fluent in both languages but, contrary to the other translator, they were familiar with the terminology used in the questionnaire. The translators’ work resulted in the development of two Polish language versions of BHCKS (T1 and T2).

**Phase II**. Based on the two translations, one questionnaire version was developed (T3). Both translators, a third translator and members of the research team all participated in the questionnaire’s development.

**Phase III**. The agreed questionnaire version was back-translated by two independent translators with the same characteristics as the ones described in Stage I. None of the translators knew the original BHCKS version. The translators’ work resulted in the development of two back-translations into English (T4 and T5).

**Phase IV**. All five versions of the questionnaire (T1-T2-T3-T4-T5) were evaluated by a multi-disciplinary team of experts whose role was to achieve trans-cultural equivalence between the original BHCKS and BHCKS_P. The expert panel consisted of an expert in statistical validation methods, and all members of the research team and the five translators involved in the earlier stages of the process. The members of the expert panel reached a consensus and developed the final version of BHCKS_P, ready for validation.

### Data analysis

In order to evaluate the psychometric properties of BHCKS_P, we assessed: content validity (content validity index), item analysis (discrimination index and floor/ceiling effects), internal consistency (Cronbach’s alpha), theoretical relevance (exploratory and confirmative factor analysis), convergent and known-groups validity.

In order to determine content validity, calculated item-level content validity index (ICVI) and scale-level content validity index (S-CVI) were used [26]. Seven experts were asked to express their opinions in a four-point Likert scale (1 = not relevant, 2 = somewhat relevant, 3 = quite relevant, 4 = highly relevant) on individual items in the context of their significance for hepatitis C knowledge. The group of experts included specialists of the Polish Association for Hepatology, the Polish Society of Epidemiology and Physicians of Infectious Diseases. The group also included internal medicine physicians, infectious disease physicians, surgeons and public health and epidemiology specialists. A CVI of more than 0.80 was interpreted as indicating content validity [26].

Item analysis applied to the discrimination of the values of indices (inter-item correlations) identified for each item. The indices should not take negative values. Moreover, the collected data were examined for occurrence of floor or ceiling effects. Following Terwee, et al. [27], it was assumed that floor or ceiling effects do not occur if no more than 15% of study participants reach the lowest or highest possible score respectively.

An analysis of BHCKS_P internal consistency was conducted based on a formula proposed by Cronbach [28]. The internal consistency threshold was assumed as satisfactory for Cronbach’s alpha greater than 0.750 [29]. Guttman’s method and the Spearman-Brown formula were used as an additional analytical approach for estimating BHCKS_P’s reliability. The Spearman-Brown formula is based on the correlation between results of two random parallel halves of the scale [30].

One-dimensionality for BHCKS_P was determined by principal component analysis. The scale which meets the Kaiser criterion (eigenvalue exceeds the value 1 only once) was considered as one-dimensional, and it was assumed that the degree of reproducibility of the indicator variables by the first principal component should exceed 40% [31].

Exploratory factor analysis (EFA) with direct oblimin rotation was used to evaluate construct validity. It was assessed whether the BHCKS_P structure was a single-item structure, which should correspond to the structure of the original BHCKS version. The number of items was distinguished based on two criteria: Kaiser [31] (own value) and Cattell [32] (scree plot). In order to determine which items would be included in their respective factors, it was decided a priori to include items that loaded at more than 0.40 on one factor. The minimum recommended sample size is ten subjects per item.

Confirmatory factor analysis (CFA) was used to estimate the accuracy of the adaptation of the obtained results vis-à-vis the imposed structure resulting from theoretical assumptions or another structure resulting from EFA. The expected values of recommended indices were as follows: χ2 divided by the degrees of freedom (chi-square/df ratio) ≤ 3.00; the Root Mean Squared Error of Approximation (RMSEA) < 0.08; the Comparative Fit Index (CFI) and the Tucker-Lewis Index (TLI) > 0.90 [33]. The value of AIC is used to compare two different models, whereby a model with a lower AIC value is preferred [34]. The calculations for EFA and CFA were conducted using the Mplus version 7.0 software.

Convergent validity was estimated by determining Spearman’s rho coefficient of correlation between the total BHCKS_P and the self-assessment of the study participants’ knowledge of HCV. The self-assessment was determined by the participants on a 10-point Visual Analogue Scale (VAS) ranging from 1 (complete lack of knowledge) to 10 (extremely high level of knowledge).

Known-groups validity was estimated based on the assessment of inter-group differences. A comparative analysis of the following four groups was carried out to that end: patients, nursing and medical students, healthcare workers. An assumption was made that healthcare workers should represent the highest knowledge level while patients the lowest. With regard to the number of members in a group, a comparison was made by means of the Kruskal–Wallis one-way analysis of variance with multiple comparisons using Dunn’s test. The effect size for the observed differences was estimated using eta-squared (η2), whereby the difference size was assumed as: small (η2: 0.010–0.039), average (η2: 0.040–0.110) or large (η2 > 0.110) [35].

Statistical calculations were performed using the statistical package IBM^®^ SPSS^®^ Statistics, version 23. For all analyses, a P-level of < 0.05 was considered statistically significant.

## Results

### Content validity

The I-CVI result for one item (BHCKS_10: some treatments for hepatitis C, such as interferon, can cause depression as a side effect in some patients) was below the assumed threshold of 0.80 (Table 2). The panel of experts did not recommend including the item in the validated BHCKS_P version. After excluding BHCKS_10, the mean result for the rest I-CVI was 0.91. The value of S-CVI obtained in this way was on a satisfactory level. A decision was made to submit an 18-item scale for validation.

**Table 2:** Ratings 19-item scale by seven experts: Items rated 3 or 4 on a 4-point relevance scale I-CIV—Item-level content validity index.

Item	Experts							Experts in Agreement	I-CVI
	A	B	C	D	E	F	G		
BHCKS_1	+	+	+	+	+	+	+	7	1.00
BHCKS_2	+	+	+	+	+	+	+	7	1.00
BHCKS_3	–	+	+	+	+	+	+	6	0.86
BHCKS_4	–	+	+	+	+	+	+	6	0.86
BHCKS_5	+	+	+	+	+	+	+	7	1.00
BHCKS_6	+	+	+	+	+	+	+	7	1.00
BHCKS_7	+	+	+	+	+	+	–	6	0.86
BHCKS_8	+	+	+	+	–	+	+	6	0.86
BHCKS_9	+	+	–	+	+	+	+	6	0.86
BHCKS_10	–	+	+	+	–	–	–	3	0.43
BHCKS_11	+	+	+	+	+	+	+	7	1.00
BHCKS_12	–	+	–	–	–	+	+	3	0.43
BHCKS_13	–	–	+	+	+	+	+	5	0.86
BHCKS_14	+	+	+	+	+	+	+	7	1.00
BHCKS_15	+	+	+	+	–	+	+	6	0.86
BHCKS_16	+	+	+	+	+	+	+	7	1.00
BHCKS_17	+	+	+	+	–	+	+	6	0.86
BHCKS_18	+	+	+	+	+	+	+	7	1.00
BHCKS_19	+	+	+	+	+	+	+	7	1.00
Proportion relevant	73.7	94.7	89.5	94.7	73.7	94.7	89.5		

I-CIV—item-level content validity index.

### Item analysis and internal consistency

The mean result obtained for the 18-item BHCKS_P in the studied group was 13.7 (SD = 3.15, CV = 23.0%) at the minimum value of 5.0 and the maximum of 18.0. The score revealed a minor left-hand-side asymmetry (skew = −0.48) and a lack of compliance with normal distribution (Shapiro–Wilk test, W = 0.943, P<0.001). Zero SD was not observed for any of the items. The lowest score was noted in 0.41% of the cases and the highest in 10.16% of the cases. The quoted results suggest a lack of floor or ceiling effects.

A negative value of inter-item correlation was not observed for any of the items (Table 3). Cronbach’s alpha coefficient for the 18-item scale amounted to 0.753, i.e. it was above the assumed 0.75 threshold. The split half reliability was also high—the estimated Guttman split half reliability coefficient value amounted to 0.858 (correlation between the halves—r = 0.751).

**Table 3:** Descriptive statistics and inter-item correlation for 18-item scale.

Item	Mean	Standard deviation	Inter-item correlation
BHCKS_1	0.67	0.47	0.623
BHCKS_2	0.55	0.50	0.159
BHCKS_3	0.95	0.22	0.017
BHCKS_4	0.87	0.34	0.339
BHCKS_5	0.73	0.44	0.277
BHCKS_6	0.81	0.39	0.296
BHCKS_7	0.86	0.35	0.058
BHCKS_8	0.96	0.21	0.180
BHCKS_9	0.50	0.50	0.032
BHCKS_11	0.96	0.21	0.306
BHCKS_12	0.88	0.33	0.361
BHCKS_13	0.82	0.39	0.372
BHCKS_14	0.67	0.47	0.705
BHCKS_15	0.64	0.48	0.185
BHCKS_16	0.67	0.47	0.560
BHCKS_17	0.65	0.48	0.515
BHCKS_18	0.96	0.20	0.136
BHCKS_19	0.66	0.48	0.707

### Construct validity

The evaluation of raw data revealed that the assumptions for EFA were met. The value of the correlation matrix determinant was close to zero (0.002), while the matrix of the coefficients of correlation was a unit matrix (Bartlett’s test of sphericity, χ2 = 1372.9, df = 153, P<0.001). The Kaiser-Meyer-Olkin index (measure of sampling adequacy) amounted to 0.777, which meets the assumptions for the parameter (> 0.500).

**Fig 1:**
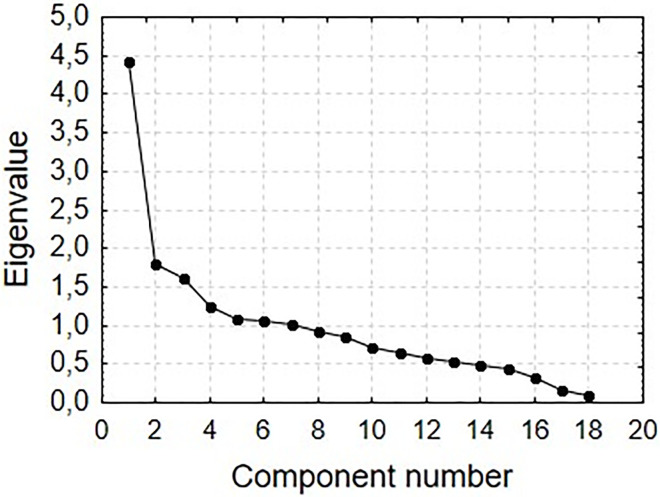
Cattell scree plot for the components extracted from data.

**Fig 2:**
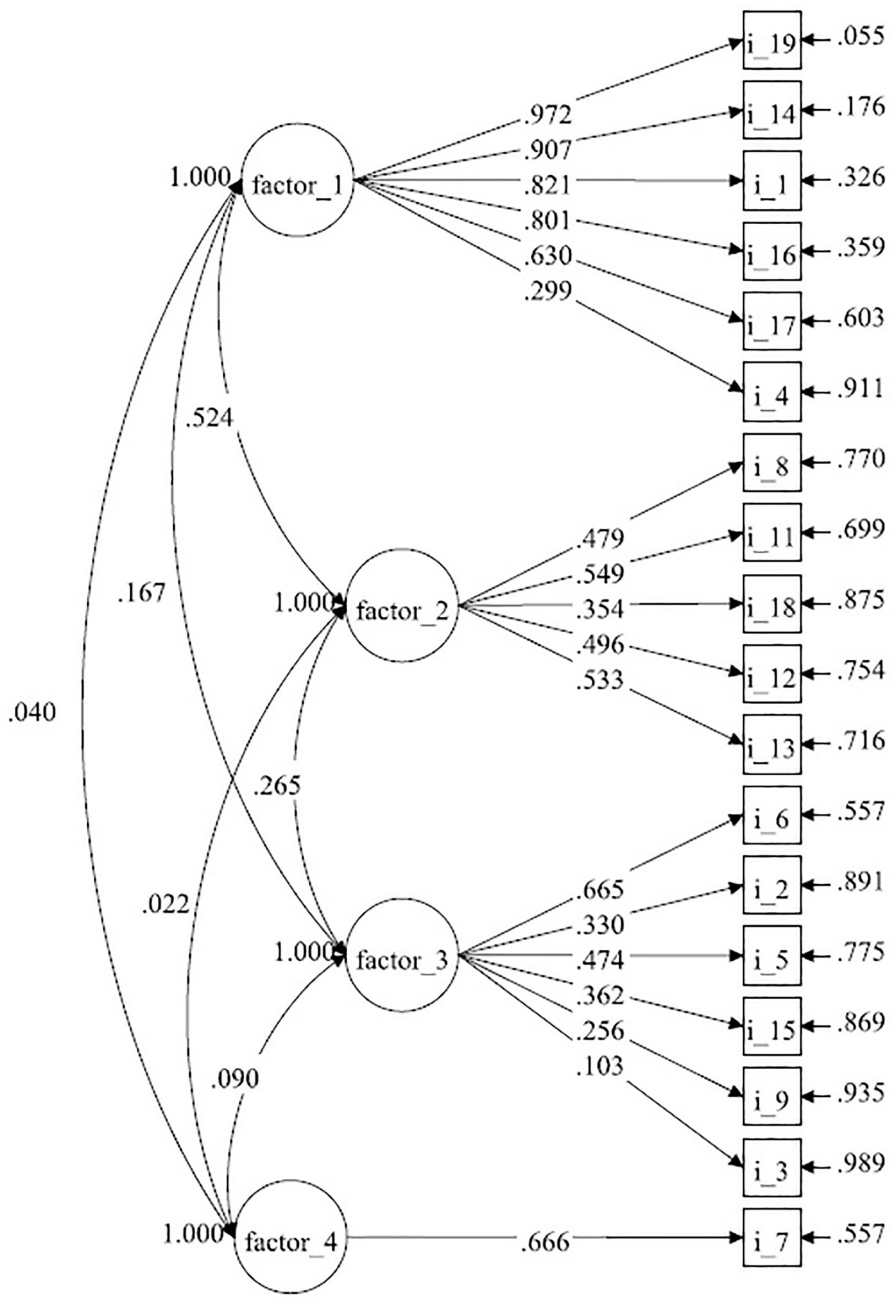
Structure of the Polish version od the Brief Hepatitis C Knowledge Scale (BHCKS). Correlations between latent variables and items are represented with arrows. The number next to the items indicates how much variance was explained in the item.

In the first EFA test, 18 items were distributed among four factors according to the Kaiser criterion, which explained a total of 50.4% of the general variance. The course of the scree plot (Cattell criterion, Fig 1) suggested a three-factor solution (the total explained variance amounted to 43.5%). Such an arrangement of factors did not comply with the original concept of BHCKS one-dimensionality. A comparison of one-dimensional goodness-of-fit and two-, three- or four-dimensional model for the collected data was carried out by means of CFA. The results for the original one-dimensional version BHCKS were less satisfactory than the two-, three- or four-dimensional version BHCKS_P (Table 4). As a result of the analysis, the ratio of chi-square statistic to degrees of freedom (χ2/df) was found to be 1.664 for the four-dimensional model (χ2 = 144.760, df = 87). The RMSEA was 0.054 (90% CI [0.038–0.069]). The TLI was 0.920 and the CFI value was 0.954. Having higher CFI and TLI values over 0.90 means that that model has a good fit. Therefore, we consider our four-factor model as confirmed (Fig 2).

**Table 4:** Goodness-of-fit of models to collected data.

Number of dimensions	χ^2^/df ratio	RMSEA (90% CI)	CFI	TLI	AIC
1	2.840	0.089 (0.079; 0.100)	0.804	0.778	2550.3
2	2.434	0.079 (0.067; 0.090)	0.867	0.827	2488.2
3	1.965	0.065 (0.051; 0.078)	0.922	0.884	2433.4
4	1.664	0.054 (0.038; 0.069)	0.954	0.920	2407.7

RMSEA—Root Mean Square Error of Approximation, CFI—Comparative Fit Index, TLI—Tucker-Lewis Index, AIC—Akaike Information Criterion, 90% CI—90% confidence interval.

### Convergent validity

An analysis revealed that the total score obtained by the study participants examined with the 18-item BHCKS_P was statistically significantly correlated with the self-evaluated level of knowledge of HCV (rho = 0.44, t = 7.195, P<0.001). The result confirmed convergent validity of the scale.

### Known-groups validity

**Fig 3:**
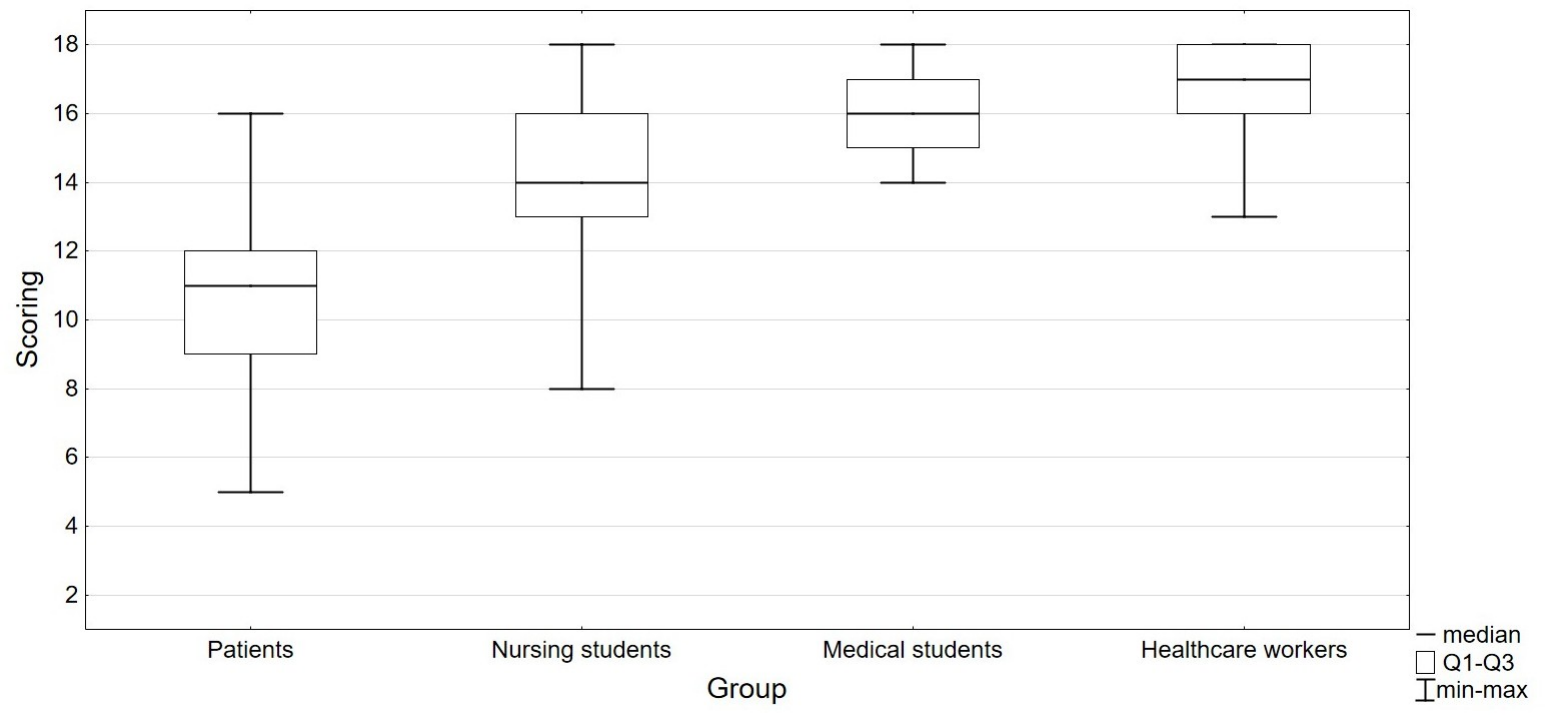
Results of the analysis of inter-group differences.

A comparative analysis of the studied groups revealed that there were statistically significant differences in the knowledge of HCV (Kruskal–Wallis test, H = 158.9, P<0.001, η2 = 0.644) (Fig 3). An evaluation by means of multiple comparisons in pairs showed that there were significant differences in the knowledge level between the group of patients and the group of nursing students (Mdn: 14.0 vs 11.0, z = 7.713, P<0.001), and between students of medicine (Mdn: 16.0 vs 11.0, z = 0.339, P<0.001) and healthcare workers (17.0 vs 11.0, z = 11.447, P<0.001). Moreover, significant differences were observed between the groups of students of nursing and medicine (Mdn: 14.0 vs 16.0, z = 3.646, P = 0.002) and healthcare workers (Mdn: 14.0 vs 17.0, z = 4.117, P<0.001). No significant differences in the knowledge level between the students of medicine and healthcare workers were observed (z = 0.377, P = 1.000).

## Discussion

The completed validation suggests good BHCKS_P psychometric characteristics with the internal consistency, convergent and known-groups validity. Similar to other validation studies, however, some differences versus the original BHCKS version were observed. First of all, based on an opinion of the expert panel, the following statement was removed from the original BHCKS version: ‘Some treatments for hepatitis C, such as interferon, can cause depression as a side effect in some patients’ (BHCKS_10). According to the experts, the statement is no longer valid in light of current guidelines on HCV treatment in Poland and worldwide (CVI-S < 0.80). Nowadays, the majority of HCV cases can be treated with direct-acting antivirals (DAA). The interferon and ribavirin treatment pattern is no longer commonly used for treating HCV-infected patients [36].

It can then be assumed that the items mentioned above are not characterised by sufficient diagnostic power. In relation to the above, including the item in BHCKS_P could result in reduced validity of measurement. In order for the Polish version of the questionnaire to meet the content validity criterion, a decision was made to limit BHCKS_P to 18 items.

Another significant difference between BHCKS_P and the original BHCKS version was a divergence in one-dimensionality. In the evaluation of the factor structure of BHCKS_P, the Kaiser criterion revealed a four-item structure of the scale, while Balfour, et al. [21] claimed that BHCKS is single-factor. Since the EFA results obtained by the authors suggested a four-factor solution, the structure was compared with a single-factor arrangement. CFA confirmed that a four-item BHCKS_P structure was a better solution than a single-factor one. The differences in the factor structure described above will not affect the results obtained during measurement with BHCKS_P, if the total score calculated for the whole scale is used exclusively.

A BHCKS_P structure different from the one assumed theoretically is evidence of the limited construct validity of the scale. This could be caused, for instance, by the reduction in the number of items as a result of the content validity evaluation procedure described above. Moreover, the difference observed in the scale structure can be partly attributed to culture differences. The obtained results suggest the need to use BHCKS_P only in the 18-item version, with no division into subscales separated during a factor analysis.

The internal consistency of BHCKS_P measured with Cronbach’s alpha coefficient turned out to be correct and exceeded the recommended value of 0.750 [29]. Positive values of inter-item correlation were observed for all items, which confirms the good differentiation of knowledge of HCV among the studied individuals subjected to assessment with BHCKS_P. Good level of reliability of BHCKS_P in the scope of internal consistency is the condition of trust in the obtained measurements taken in the future using this tool. This is connected with a low level of sampling errors, which do not exceed 25% [37].

In the course of BHCKS reliability measurement, Balfour, et al. [21] estimated the scale absolute stability by means of repeated measurement with the same tool (test-retest). The results obtained in the first test were then compared with the re-test results. The Pearson’s correlation was the reliability measure [21]. The method suffers from an error related to the need for performing a re-test in a distant time perspective [38]. The study participants are tested twice with the same tool. The questionnaire presented during the second session is not new for the study participants, as it was also used in the first measurement. The results of the reliability analysis can thus be affected by two basic factors: memory and learning. If one is tested twice with the same tool, it cannot be excluded that their knowledge or skills level has changed in the period between the measurements [30]. That is why the authors of the presented study resigned from reliability evaluation with the test-retest method and proposed an alternative evaluation using correlation evaluation, not between the repeated measurements but between the results of a single study divided into random and equivalent halves [30]. Reliability measured with the Guttman split half reliability coefficient was high and exceeded the value of 0.850. The obtained result was characterised by higher split half reliability when compared with the reliability calculated using Cronbach’s formula.

With regard to the fact that in Poland there is no validated tool other than BHCKS_P to evaluate the knowledge of HCV, the obtained BHCKS_P measurement results could not be referred to the results of measurements with the gold standard. This limited the possibility for a full evaluation of the external criterion validity. In relation to the above, a decision was made to choose an intermediate evaluation of the validity by correlating the results obtained by means of BHCKS_P with self-evaluation of the study participants’ knowledge in a 10-point VAS. The results of the correlation analysis confirmed a positive relationship between the scores obtained in both measurements. In spite of limitations related to the lack of a gold standard, it can be concluded that BHCKS_P measurement is characterised by an evaluation correlating with the external criterion, which is confirmed by the tool’s convergent validity.

A known-groups validity evaluation varied slightly from the one proposed by Balfour, et al. [21]. An analysis of BHCKS_P differentiating capacity was modified taking into account the evaluation of differences between the four groups of respondents participating in the study. According to the assumptions, it was demonstrated that healthcare workers had the highest knowledge level, followed by students of medicine and nursing. The lowest scores were observed in the patients’ group. The ability to differentiate the results of the measurements performed with BHCKS_P in the groups was very high (η2 > 0.110). The results mentioned above confirm good BHCKS_P properties with known-groups validity.

Despite the fact that in the group of healthcare workers the knowledge levels were relatively high, there were a few cases with unsatisfactory levels of knowledge of HCV. This result is not, however, surprising as similar gaps in knowledge were also observed by other researchers in the healthcare workers group [21, 39–42]. This insufficient knowledge level among healthcare workers as well as patients and nursing and medicine students confirms the necessity to identify the knowledge gaps regarding HCV, which is very important when developing new education programmes in this field. This is confirmed, among others, by studies by Mencl et al. (2000) [41] and Coppola at al. (2004) [42, 43] as well as Gupta et al. (2000) [44].

The results of BHCKS_P psychometric analysis allow the assumption that the scale can be used in practice as a tool for assessing the knowledge of HCV among patients, students and healthcare workers. The assessment results can be used for the evaluation of study curricula, which include mandatory education on diagnostics and treatment of infectious disease. Moreover, individual results can be used satisfactorily for self-evaluation and as a tool to measure the effectiveness of educational programmes (pre- and post-training tests). The questionnaire can also be used for designing individual educational programmes, indicating areas of special focus during the educational process (graduate and post-graduate education). The short form (18 items) of BHCKS_P is definitely an advantage, because it only takes a few minutes to fill in the questionnaire. BHCKS_P can be easily adapted for sharing in an electronic format, while calculation and interpretation of the results can be automated.

The main limitation of the validation study was the lack of compliance evaluation of the results of measurements carried out with BHCKS_P with the results of measurements obtained with another tool, i.e. gold standard questionnaire, whose theoretical construct was similar to BHCKS. It was not possible to carry out such an analysis due to the lack of a Polish tool dedicated to the evaluation of knowledge of HCV. It is necessary to carry out general Polish studies using BHCKS_P in order to identify the standards. In addition, further validation studies should assess the diagnostic ability of the Polish BHCKS, by performing research using the tool in educational situations (i.e. pre- and post-training tests).

## Conclusions

The results of a psychometric analysis of the Polish BHCKS version confirm good quality of the tool. For its reliability and validity, BHCKS_P is comparable with the original English version. The obtained results of the measurement provide information about the studied person based on the total score. BHCKS_P reveals good sensitivity to inter-group discrimination and is characterised by internal consistency as a uniform scale.

## Supporting information

S1 Data (DOCX)
