# Supplementary material for: Translation and validation study of the Polish version of the Brief Hepatitis C Knowledge Scale
Source: PLoS One. 2020 Jul 9;15(7):e0235764. doi: 10.1371/journal.pone.0235764 (PMC7347207; doi:10.1371/journal.pone.0235764)
Supplement: S1 Data — (DOCX) [file pone.0235764.s001.docx]

**Polska wersja językowa skali oceny wiedzy**

**na temat wirusowego zapalenia wątroby typu C (BHCKS_PL)^[[1]](#footnote-1)^**

| **Numer** | **Treść stwierdzenia** | **Prawda** | **Fałsz** | **Nie wiem** |
| --- | --- | --- | --- | --- |
| BHCKS_1 | Osoby z WZW C mogą bezpiecznie dzielić się z innymi osobami swoimi szczoteczkami do zębów i maszynkami do golenia | □ | □ | □ |
| BHCKS_2 | Osoby z WZW C mogą bezpiecznie stosować wszelkie ziołowe produkty lecznicze | □ | □ | □ |
| BHCKS_3 | Spożywanie alkoholu przez osoby z WZW C może prowadzić do uszkodzenia wątroby | □ | □ | □ |
| BHCKS_4 | Osoby, u których w Polsce, przed 1992 r., przeprowadzono transfuzję preparatów krwiopochodnych, mogły zostać zakażone wirusowym zapaleniem wątroby typu C | □ | □ | □ |
| BHCKS_5 | Szczepionka przeciw WZW C może być stosowana w ramach zapobiegania nowym zakażeniom tym wirusem | □ | □ | □ |
| BHCKS_6 | Osoby z przewlekłym WZW C powinny być szczepione przeciw WZW A i B | □ | □ | □ |
| BHCKS_7 | Badania wskazują, że ponad 60% osób przyjmujących dożylnie narkotyki "używanymi igłami" jest zakażonych WZW C | □ | □ | □ |
| BHCKS_8 | Osoby zakażone WZW C przez wiele lat mogą nie być świadome zakażenia | □ | □ | □ |
| BHCKS_9 | U osób przyjmujących donosowo kokainę istnieje ryzyko transmisji WZW C poprzez stosowanie wspólnych słomek, zwiniętych banknotów itp. | □ | □ | □ |
| BHCKS_11 | Stosowanie nowych, nigdy wcześniej nieużywanych igieł, strzykawek i innego sprzętu zmniejsza ryzyko zakażenia WZW C | □ | □ | □ |
| BHCKS_12 | Dzieci matek zakażonych WZW C mogą zostać zakażone podczas porodu | □ | □ | □ |
| BHCKS_13 | Do zakażenia WZW C może dojść podczas kontaktu seksualnego | □ | □ | □ |
| BHCKS_14 | WZW C może rozprzestrzeniać się poprzez kaszel i kichanie | □ | □ | □ |
| BHCKS_15 | Skuteczna terapia przeciwwirusowa HCV może prowadzić do całkowitego wyeliminowania wirusa z krwi pacjenta | □ | □ | □ |
| BHCKS_16 | WZW C może rozprzestrzeniać się poprzez używanie wspólnych naczyń kuchennych (kubków, talerzy, sztućców, itp.) | □ | □ | □ |
| BHCKS_17 | Osoby po skutecznej terapii przeciwwirusowej HCV i eradykacji wirusa nie mogą ponownie być zakażone tym wirusem | □ | □ | □ |
| BHCKS_18 | WZW C można zarazić się podczas wykonywania zabiegu tatuażu lub piercingu | □ | □ | □ |
| BHCKS_19 | Do zakażenia WZW C może dojść poprzez uścisk dłoni lub przytulenie | □ | □ | □ |

**Klucz poprawnych odpowiedzi:**

Prawda: 3, 4, 6, 7, 8, 9, 11, 12, 13, 15, 18

Fałsz: 1, 2, 5, 14, 16, 17, 19

**Interpretacja:**

Za każde poprawne wskazanie odpowiedzi przyznaje się 1 punkt.
Każde niepoprawne wskazanie lub zaznaczenie opcji „nie wiem” przyznaje się 0 punktów.
Maksymalna możliwa punktacja to 18 punktów.

Im wyższa jest suma punktów zyskana przez badanego tym wyższy jest poziom jego wiedzy na temat wirusowego zapalenia wątroby typu C.

Dla skali nie wyznaczono norm.

1. Original English version of The Brief Hepatitis C Knowledge Scale developed by Balfour L, et al. Increasing public awareness about hepatitis C: development and validation of the brief hepatitis C knowledge scale. Scand J Caring Sci. 2009; 23(4): 801-8. doi: 10.1111/j.1471-6712.2008.00668.x. [↑](#footnote-ref-1)
